# The Association between Door-to-Balloon Time of Less Than 60 Minutes and Prognosis of Patients Developing ST Segment Elevation Myocardial Infarction and Undergoing Primary Percutaneous Coronary Intervention

**DOI:** 10.1155/2017/1910934

**Published:** 2017-04-04

**Authors:** Fu-Cheng Chen, Yan-Ren Lin, Chia-Te Kung, Cheng-I Cheng, Chao-Jui Li

**Affiliations:** ^1^Department of Emergency Medicine, Kaohsiung Chang Gung Memorial Hospital, Chang Gung University College of Medicine, Kaohsiung, Taiwan; ^2^Department of Emergency Medicine, Changhua Christian Hospital, Changhua, Taiwan; ^3^School of Medicine, Kaohsiung Medical University, Kaohsiung, Taiwan; ^4^School of Medicine, Chung Shan Medical University, Taichung, Taiwan; ^5^Division of Cardiology, Department of Internal Medicine, Kaohsiung Chang Gung Memorial Hospital, Chang Gung University College of Medicine, Kaohsiung, Taiwan

## Abstract

*Background.* The study aimed to verify the effect of primary percutaneous coronary intervention (PPCI) with <60 min door-to-balloon time on ST segment elevation myocardial infarction (STEMI) patients' prognoses.* Methods.* Outcomes of patients receiving PPCI with door-to-balloon time of <60 min were compared with those of patients receiving PPCI with door-to-balloon time 60–90 min.* Result.* Totally, 241 STEMI patients (191 with Killip classes I or II) and 104 (71 with Killip classes I or II) received PPCI with door-to-balloon time <60 and 60–90 min, respectively. Killip classes I and II patients with door-to-balloon time <60 min had better thrombolysis in myocardial infarction (TIMI) flow (9.2% fewer patients with TIMI flow <3, *p* = 0.019) and 8.0% lower 30-day mortality rate (*p* < 0.001) than those with 60–90 min. After controlling the confounding factors with logistic regression, patients with door-to-balloon time <60 min had lower incidences of TIMI flow <3 (aOR = 0.4, 95% CI = 0.20–0.76), 30-day recurrent myocardial infarction (aOR = 0.3, 95% CI = 0.10–0.91), and 30-day mortality (aOR = 0.3, 95% CI = 0.09–0.77) than those with 60–90 min.* Conclusion.* Door-to-balloon time <60 min is associated with better blood flow in the infarct-related artery and lower 30-day recurrent myocardial infarction and 30-day mortality rates.

## 1. Introduction

The American Heart Association guidelines suggest primary percutaneous coronary intervention (PPCI) as the preferred treatment for ST segment elevation myocardial infarction (STEMI) patients [1]. For nontransfer patients, PPCI should be performed within 90 min of arrival at a hospital. The door-to-balloon time is strongly associated with the likelihood of survival and is an accepted measure of care quality [2, 3]. Multiple strategies have been utilized to reduce the door-to-balloon time [4–6]. However, recently, some studies have reported that significantly shortened door-to-balloon time may not improve the mortality rate of STEMI patients who are undergoing PPCI [7, 8]. This finding raises the question of whether shortening of the door-to-balloon interval is necessary. Since the 2012 European Society of Cardiology guidelines suggested that the goal should be to achieve a door-to-balloon time of less than 60 min of presentation in PPCI-capable institutions [9], few studies have focused on the effect of <60 min door-to-balloon time on the outcome of STEMI patients. Recently, Wang et al. (2016) reported that <60 min door-to-balloon time is associated with better survival rates in younger STEMI patients undergoing PPCI than in their elderly counterparts [10]. However, this study also included patients undergoing PPCI with >90 min door-to-balloon time, which might have influenced the results. Our study focused on the difference between door-to-balloon times of <60 min and 60–90 min, which could help to determine whether further shortening of the door-to-balloon time is necessary.

## 2. Methods

### 2.1. Study Design

This retrospective study was approved by the Chang Gung Medical Foundation Institutional Review Board. All data in the patient and physician records were anonymized and deidentified. The research protocol was approved by the Ethics Committee, and a waiver of informed consent was granted.

### 2.2. Study Setting and Participants

This study was conducted in a 3000-bed tertiary referral medical center located in Kaohsiung City in Southern Taiwan. Over 130,000 patients visit the emergency department (ED) annually. More than 150 STEMI patients (including transfer and nontransfer patients) are treated each year, nearly all of whom receive PPCI as a reperfusion therapy. STEMI patients receiving PPCI between January 1, 2011, and December 31, 2014, were included in this study. Patients aged ≥18 years who arrived at the ED within 12 h of symptom onset and met the diagnostic criteria of acute STEMI assessed through electrocardiogram (ECG) (ST segment elevation > 1 mm in two contiguous limb leads and 2 mm in precordial leads or the presence of new-onset left bundle branch block) [11] and coronary artery disease confirmed by PPCI were included. We excluded patients with >90 min door-to-balloon time and those with prolonged cardiopulmonary resuscitation in the ED because of their expected poor outcomes. Patients referred from other hospitals were also excluded.

### 2.3. Study Protocol

PPCI was performed in accordance with the protocol of the study hospital [12–14]. A transradial artery approach using 6F Kimny guiding catheter (Boston Scientific, One Scimed Place, Maple Grove, MN, USA) was utilized for both coronary arterial occlusion diagnosis and PPCI. Intra-aortic balloon pump (IABP) support was performed through the femoral artery in patients experiencing acute pulmonary edema associated with unstable condition or hemodynamic instability. Patients whose systolic blood pressure could not be maintained above 75 mmHg after IABP support and intravenous administration of more than 20 *μ*g/kg/min dopamine were treated with extracorporeal membrane oxygenation (ECMO). All patients received dual antiplatelet therapy with a loading dose of clopidogrel (600 mg) or ticagrelor (180 mg), each combined with aspirin (300 mg), in the emergency department, followed by treatment with a maintenance dose of the same medications. The dual antiplatelet therapy was discontinued in cases where patients experienced major bleeding. The outcomes of patients who received PPCI with door-to-balloon time of <60 min (<60 group) were compared with those of patients who received PPCI with door-to-balloon time between 60 and 90 min (60–90 group).

### 2.4. Measures

The patient demographic and clinical information was obtained from the ED administrative database. The outcome indicators after PPCI included the left ventricular (LV) function and rates of the final thrombolysis in myocardial infarction (TIMI) 3 blood flow in the infarct-related artery, 30-day recurrent myocardial infarction (MI), and 30-day mortality. The LV function was assessed using transthoracic echocardiography. Additionally, the internal LV dimensions (i.e., end-systolic diameter [ESD] and end-diastolic diameter [EDD]) were measured based on the American Society of Echocardiography's leading-edge method using at least three consecutive cardiac cycles with the patients in the supine position. The LV ejection fraction (LVEF) was calculated as follows: LVEF (%) = [(LV EDD^3^  – LV ESD^3^)/LV EDD^3^] × 100%.

### 2.5. Statistics

For continuous variables, the data were summarized as the mean and standard deviation (SD) and analyzed using Student's *t*-test. Categorical variables were summarized as numbers and percentages, and the chi-square test was used to evaluate the associations between the outcome groups. In the multivariate analyses, binary logistic regression models were applied to assess the effect of <60 min door-to-balloon time on documented patient outcomes to adjust for the potential confounding factors. The effects were estimated in terms of adjusted odds ratios (aORs) and the corresponding 95% confidence intervals (CIs). The results were considered statistically significant if a *p* value < 0.05 was obtained (two-tailed Student's *t*-test). The statistical analysis was performed using SPSS for Windows version 12.0 (SPSS, Chicago, IL, USA).

## 3. Results

### 3.1. Patient Demographics

During the study period, the data of 345 adult patients with STEMI visiting the ED were analyzed. A total of 241 (69.9%) and 104 patients (30.1%) received PPCI with door-to-balloon times <60 min and between 60 and 90 min, respectively.  Table 1 shows the baseline demographics and clinical histories of the two study groups. The baseline demographics were comparable between the two study groups.

### 3.2. Event and Procedural Characteristics

Table 2 presents the event and procedural characteristics. In the <60 group, the time from patient registration to electrocardiography examination and the time of a catheter guidewire crossing the culprit lesion in the cardiac catheterization laboratory were 3.8 and 48.4 min, respectively. In the 60–90 group, the corresponding values were 9.8 and 72.2 min, respectively. The drug selection for the dual antiplatelet therapy was similar in the two study groups. Twenty-four patients stopped receiving aspirin treatment due to bleeding (14 [5.8%] and 10 [9.6%] patients in the <60 and 60–90 groups, resp., [*p* = 0.202]). More patients presented with Killip class III or IV MI and received cardiopulmonary resuscitation, endotracheal intubation, and IABP in the 60–90 group compared to the <60 group. The differences in the incidence of pulseless ventricular tachycardia, ventricular fibrillation, and atrioventricular conduction block and ECMO use were not statistically significant between the two study groups. The numbers of occluded vessels in the two groups were also similar (Table 2).

### 3.3. Outcome

**Figure 1:**
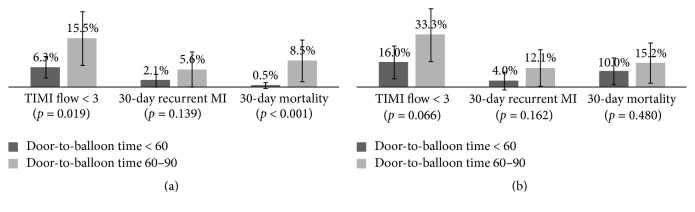
The incidence of TIMI flow < 3, 30-day recurrent MI, and 30-day mortality of patients with door-to-balloon time < 60 min and 60–90 min in Killip I and II (Figure 1(a)) and Killip III and IV (Figure 1(b)).

Overall, the LVEF was 57.1%, the final TIMI flow < 3 incidence in the infarct-related artery was 12.2%, and the 30-day recurrent MI and 30-day mortality rates were 4.1% and 4.9%, respectively. A stratified analysis was conducted considering the difference in Killip class distribution in the two study groups. No statistical difference in the LV function was found in the subgroup analysis. The mean LVEFs of Killip classes I and II patients with door-to-balloon times of <60 and 60–90 min were 58.8% and 57.8% (*p* = 0.631), respectively. Additionally, those of Killip classes III and IV patients with door-to-balloon times of <60 and 60–90 min were 53.4% and 51.0% (*p* = 0.400), respectively. However, in patients with Killip classes I and II MI (Figure 1(a)), those with <60 min door-to-balloon time had better blood flow in the infarct-related artery (9.2% fewer patients with TIMI flow < 3, *p* = 0.019) and 8.0% lower 30-day mortality rate (*p* < 0.001) than those with 60–90 min door-to-balloon time. No statistical significance was observed in patients with Killip classes III and IV MI (Figure 1(b)), although the <60 group seemed to have better outcomes than the 60–90 group. A logistic regression model analysis was conducted to simultaneously control the potential confounding factors, including age, sex, and Killip class. Patients with <60 min door-to-balloon time had lower incidence of TIMI flow < 3 (aOR = 0.4, 95% CI = 0.20–0.76) and rates of 30-day recurrent MI (aOR = 0.3, 95% CI = 0.10–0.91) and 30-day mortality (aOR = 0.3, 95% CI = 0.09–0.77) than those with 60–90 min door-to-balloon time (Table 3).

## 4. Discussion

The effect of the <60 min door-to-balloon time on the outcomes of STEMI patients has not been widely studied. One study demonstrated that the prevalence of the final TIMI flow < 3, advanced congestive heart failure, and 30-day mortality did not differ between patients with <60 min door-to-balloon time and those with >60 min door-to-balloon time [15]. This finding might be attributed to the inclusion of referral patients in the study. Thus, the treatment time may have been influenced by the transfer interval. Wang et al. (2016) reported that ≤60 min door-to-balloon time was associated with better survival rates in younger STEMI patients undergoing PPCI than in elderly patients [10]. However, this study excluded patients undergoing PPCI with >90 min door-to-balloon time. After exclusion of patients with >90 min door-to-balloon time, who might potentially have worse prognosis, our study demonstrated that the shortening of door-to-balloon time to <60 min could improve the postprocedural TIMI flow and lower the 30-day recurrent infarction and 30-day mortality rates.

The results of our study demonstrate that the most important effect of shortening the door-to-balloon time to <60 min was the lowered 30-day mortality rate. The overall mortality rate was 4.9%, which was slightly higher than the data reported by Menees et al. (2013) (3.8%) [8]. We believe that this finding might be caused by the differences in disease severity distribution. Following the stratification of the analysis, we found that the subgroup with Killip classes I and II MI showed a major difference in mortality rate, which was 8% lower in patients with <60 min door-to-balloon time than in those with 60–90 min door-to-balloon time. In fact, in patients with Killip classes III and IV MI, those with <60 min door-to-balloon time displayed 5.2% lower mortality rate than those with 60–90 min door-to-balloon time, although the difference was not statistically significant. We found that door-to-balloon time of <60 min still played an important role in patient mortality rate reduction even after controlling the potential confounding factors, including age, sex, and Killip class, using a regression model. Therefore, the decreased time interval used may have been insufficient in the recent studies reporting that further shortening of the door-to-balloon time might not improve the patient mortality rate [7, 8]. We believe that the shortening of door-to-balloon time to <60 min could improve STEMI mortality rate by excluding referral patients and controlling for disease severity.

Some studies have reported healthcare system issues and patient demographic characteristics as predictors of door-to-balloon time delay, including the need for hospital transfer, nondaytime presentation, low-volume medical units, older age, female sex, and race [16, 17]. Swaminathan et al. (2013) have highlighted some clinical issues as predictors of door-to-balloon time delay, including resuscitation for cardiac arrest, intubation for respiratory failure, difficulty in obtaining vascular access and crossing the culprit lesion, and providing consent [18], as we did in our study. More patients received cardiopulmonary resuscitation, intubation, and IABP in the 60–90 group compared to the <60 group. Interestingly, the door-to-ECG time was 6 min shorter in the <60 group than in the 60–90 group. The shortening of door-to-ECG time might be an important strategy to reduce the door-to-balloon time. ECG is a key step for STEMI diagnosis. However, one challenge in the diagnosis is that one-third of patients with MI do not experience chest pain [19] and thus are given a low acuity triage score when they present at an ED, which is associated with ECG and treatment delays [20, 21]. Such patients have increased morbidity and mortality compared with those who present with chest pain [19, 22]. One solution for this problem might be the establishment of a chief complaint-based cardiac triage protocol to streamline ECG completion and shorten the door-to-ECG time [23].

## 5. Conclusion

Our study demonstrated that <60 min door-to-balloon time is associated with better blood flow in the infarct-related artery and lower 30-day recurrent MI and 30-day mortality rates.

## Figures and Tables

**Table 1:** Baseline demographic and clinical history.

Variables	Door-to-balloon < 60 min (*n* = 241)	Door-to-balloon 60~90 min (*n* = 104)	*p* value
Age (years)	59.0 ± 12.04	62.6 ± 12.06	0.012
Male	213 (88.4%)	90 (86.5%)	0.631
Body mass index (kg/m^2^)	25.5 ± 3.68	25.0 ± 4.19	0.303
Mean artery pressure (mmHg)	105.0 ± 26.87	101.6 ± 24.24	0.278
Diabetes	73 (30.3%)	36 (34.6%)	0.428
Hypertension	152 (63.1%)	68 (65.4%)	0.682
Hyperlipidemia	139 (57.7%)	56 (53.8%)	0.510
Smoking	122 (50.6%)	42 (40.4%)	0.081
Previous myocardial infarction	21 (8.7%)	14 (13.5%)	0.180
History of PCI^*∗*^	21 (8.7%)	14 (13.5%)	0.180

^*∗*^PCI: percutaneous coronary intervention.

**Table 2:** Event and procedural characteristics.

Variables	Door-to-balloon < 60 min (*n* = 241)	Door-to-balloon 60~90 min (*n* = 104)	*p* value
Door-to-ECG time	3.8 ± 4.95	9.8 ± 9.81	<0.001
Door-to-balloon time	48.4 ± 7.99	72.2 ± 14.09	<0.001
Dual antiplatelet therapy			0.802
Clopidogrel and aspirin	180 (74.7%)	79 (76.0%)	
Ticagrelor and aspirin	61 (25.3%)	25 (24.0%)	
Killip III-IV	50 (20.7%)	33 (31.7%)	0.029
Pulseless VT/Vf^*∗*1^	20 (8.3%)	14 (13.5%)	0.140
AV conduction block^*∗*2^	17 (7.1%)	12 (11.5%)	0.168
Cardiopulmonary resuscitation	11 (4.6%)	13 (12.5%)	0.008
Endotracheal intubation	16 (6.6%)	20 (19.2%)	<0.001
Intra-aortic balloon pumping	33 (13.7%)	24 (23.1%)	0.031
Extracorporeal membrane oxygenation	6 (2.5%)	5 (4.8%)	0.261
Occlusion vessel number			0.735
One	120 (49.8%)	47 (45.2%)	
Two	55 (22.8%)	26 (25.0%)	
Three	66 (27.4%)	31 (29.8%)	

^*∗*1^Pulseless VT/Vf: pulseless ventricular tachycardia/ventricular fibrillation.

^*∗*2^AV conduction block: atrioventricular conduction block.

**Table 3:** The association between door-to-balloon time less than 60 minutes and patient outcome by logistic regression analysis.

Outcome	Door-to-balloon < 60 min		Door-to-balloon 60~90 min
	aOR	95% CI	Reference
TIMI flow < 3^*∗*^	0.4	0.20~0.76	1
30-day reinfarction	0.3	0.10~0.91	1
30-day mortality	0.3	0.09~0.77	1

^*∗*^TIMI flow < 3: thrombolysis in myocardial infarction (TIMI) flow < 3. aOR: adjusted odds ratio, adjusted for the potential confounding factors including age, sex, and Killip class. 95% CI: 95% confidence interval.

## Additional Points

Several limitations exist in this study. First, this is a single-center study with a relatively smaller sample size. Second, some confounding factors may still not be accounted for due to the retrospective nature of the study. Third, the symptom-to-balloon time is not included in the analysis of this study, which might also influence the result. Fourth, this study did not trace the patients' long-term outcomes; thus, the long-term mortality rate or quality of life was not discussed.
